# Tracing Data Journeys Through Medical Case Reports: Conceptualizing Case Reports Not as “Anecdotes” but Productive Epistemic Constructs, or Why Zebras Can Be Useful

**DOI:** 10.1007/978-3-030-37177-7_4

**Published:** 2019-12-05

**Authors:** Rachel A. Ankeny

**Affiliations:** 1 Department of Sociology, Philosophy and Anthropology & Exeter Centre for the Study of the Life Sciences (Egenis), University of Exeter, Exeter, UK, Alan Turing Institute, London, UK; 2 Department of Sociology, Philosophy and Anthropology & Exeter Centre for the Study of the Life Sciences (Egenis), University of Exeter, Exeter, UK, Alan Turing Institute, London, UK; Departments of History and Philosophy, University of Adelaide, Adelaide, SA Australia

## Abstract

Medical case reports provide an important example of data journeying: they are used to collect data and make them available for re-use to others in the field including clinicians, biomedical researchers, and health policymakers. In this paper, I explore how data journey in case reports, with particular focus on the earliest stages of the process, namely from creation and publication of case reports to the initial re-uses of them and data within them. I investigate key themes relating to case reporting and re-use, including factors which seem to smooth the path along which the data captured by a case report journey via broader citation patterns and detailed qualitative analysis of highly re-used case reports. This analysis reveals some of the key factors associated with the case reports whose data have greater amounts of journeying including publication in a general medical journal; that the data have broader implications and evidential value for topical or even urgent issues for instance in public health; and use in the case report of multiple research methods or concepts from diverse subfields. These findings along with standardization of case reporting are shown to have epistemological implications, particularly for how we understand the journeying of data.

## Introduction

Data never stand on their own: they are gathered and become accessible via different forms of “packaging” (Ankeny 2010; Leonelli 2010, 2016) and travel over space and time. These journeys associated with their use and re-use in various contexts shape how they are understood, interpreted, and subsequently utilized. The issues associated with curation, imposing ontologies, and establishing metadata via online databases are well recognized, including the resulting epistemological limitations (Leonelli 2016). Standardization of data is a critical part of such processes and is extremely complex even where the data in question are relatively simple (such as genomic sequencing data in organism-based databases, see Leonelli and Ankeny 2012), let alone in fields where data are highly heterogeneous (e.g., in this volume see the chapters by Halfmann, Parker, Ramsden, and Wylie).

Clinical research is a domain of scientific practice where data often are extremely complex and collected in highly variable and non-standardized ways. The complexities associated with the data collected typically arise not because of the content of the data but because of our (high) level of interest in the details and the mixture of subjective and objective types of information in play whenever the main focus is on humans and particularly patients. Various types of data can be more easily standardized than others, for instance those collected in randomized controlled trials (RCTs), which can be easily aggregated using meta-analysis or similar. However other types of clinical data are much more diverse in terms of quantity, quality, provenance, means of production, attached metadata, and so on.

Medical case reports are a particularly striking example: among other purposes, they are used to collect data and make them available for re-use by others including clinicians, biomedical researchers, and health policymakers (for other uses, see e.g. Ankeny 2010, 2014, 2017a). Case reports are an ideal focus for exploring of how data “journey” at their earliest stages. They do not tend to cover great distances in any literal sense, but instead move from one context to another and thus allow exploration and development of understanding via application in new domains.

In this paper, I explore how data journey in case reports with particular focus on the earliest stages of the process, namely from creation and publication of case reports to the initial re-uses of them and data within them. Following presentation of background on medical case reports, I investigate key themes relating to case reporting and re-use, including factors that seem to smooth the path of the journey along which the data travel, via broader citation patterns and detailed qualitative analysis of highly re-used case reports. This analysis reveals some of the key factors associated with the valuing of data captured by case reports by those in the broader biomedical and health communities, as well as allowing reflections on how and when case reports are most useful and how standardization of case reporting might support the journeying of data.

As in the historical sciences, much of what is contained in medical case reports is contingent. In both fields, narratives are particularly useful ways of accounting for contingent outcomes by providing detailed data relating to them since narratives allow capture of rich descriptions that permit the envisioning of alternative possibilities or relationships.^1^ Medical case reporting also involves processes of toggling back and forth between individual instances (observations on a specific patient) and the generalizations that might follow from them, if only implicitly.^2^ Thus this account also has relevance for the historical sciences and other sciences which depend closely on contingent and local observational data.

## Background: What Are Medical Case Reports?

Medical case reports have been utilized for centuries to record and disseminate unusual presentations of illness that cannot be readily identified or that do not easily map onto recognized clinical conditions. Using a detailed narrative format,^3^ they outline the diagnosis, treatment, and outcomes typically of a single patient (or a small series of patients) with a focus on practice-based observations and clinical care, rather than the results of RCTs or other experimental methodologies. One goal of case reporting is to capture data on specific instances of phenomena including many details that may not be immediately relevant, but may prove to be: the data do not have an immediate or definite purpose or target, but are collected because of their potential and future evidential value, which often is not clear when the case report is written or published. Thus these data (and the case report itself) are made available for re-use over time as subsequent instances of similar illnesses arise or as the data within the case report becomes relevant for another purpose, and so can be systematically combined into larger datasets and hence journey beyond their original domain.

Unlike RCTs or similar, the data typically contained in case reports are highly non-standardized, and include a mixture of quantitative and qualitative information. Accordingly, they are treated as one of the lowest types of evidence in the hierarchy associated with the evidence-based medicine (EBM) movement (Nissen and Wynn 2012). Some critics even have argued that highlighting the rare and unusual (termed by them “anecdotal”) is dangerous, because they can lead clinician-readers to mistaken interpretations about what they are seeing in seeing in their patients and what is likely (Hoffman 1999) or that they rely on specious claims made by clinicians who wish to get published but without doing the required research (McGee 2006). It also has been documented that case reports do not receive nearly as many citations as meta-analyses or randomized controlled trial (Patsopoulos et al. 2005), and are read far less often (Leopold 2015). Hence some journals have limited the number of case reports that they publish, imposed much more detailed and stringent guidelines, or even stopped publishing them altogether. From their point of view, to borrow a phrase, “the plural of anecdote is not data” (Leopold 2015, 3074).

Advocates of case reports defend their use for particular types of purposes (e.g., Godlee 1998; Vandenbroucke 1999, 2001; Wright and Kouroukis 2000; Carey 2006; Smith 2008; Smalheiser et al. 2015; Rison et al. 2017): first, they can serve as the basis of hypotheses and direct future clinical research especially about the efficacy of interventions, side effects of certain treatments or drugs, and aspects of clinical practice relating to individualized treatment (see Ankeny 2014, 2017a). Case reports have proven useful for identifying adverse and beneficial effects and recognizing new or rare diseases or unusual manifestations of common diseases; an oft-cited example of the success of case reporting is the recognition of the relationship between use of the drug thalidomide by pregnant women and congenital abnormalities in newborns (McBride 1961; Lenz 1962). Case reports can serve as a type of evidence even in EBM when used in the appropriate manner (Jenicek 2001) and can also be useful in clinical education (Cabán-Martinez and García-Beltrán 2012), particularly given the dominance of problem-based learning approaches in medicine. Finally, there is some evidence that case reports can make significant contributions to medical research planning (Albrecht et al. 2005).

Case reports account for a rapidly growing number of medical publications and an increasing number of dedicated journals in recent years, with at least 160 case reports journals from 78 publishers documented as of mid-2015 (for a useful summary, see Akers 2016, Table 1 available online), with observers commenting that there has been a “renaissance of the case reporting literature” (Smalheiser et al. 2015, 171). More generally in the field of medicine considered as a whole, the number of MEDLINE-listed case reports is said to substantially exceed the number of published clinical studies (Kiene et al. 2013). The newer journals tend to be open access and range from having a focus on general medical issues to accounts of case reports in more specialized subfields. Unfortunately, predatory publishing practices are particularly rampant among case report journals (Akers 2016), with nearly 50% of publishers engaging in questionable publishing practices. In addition, few have impact factors, in part because of the infrequency with which case reports are cited, but nearly half of the journals (as of mid-2015) are indexed in PubMed (Akers 2016), making them accessible to clinicians and researchers and for analysis of the types performed in the current paper.

Unlike other parts of medical training and publication (e.g., differential diagnostic processes or mortality and morbidity reporting, see Bosk 1979), the processes of recording this type of historical data generally have not been made consistent or standardized. Thus case reports have been viewed by many within the field as insufficiently rigorous for aggregation for data analysis which would be rigorous enough to inform research design and allow data to journey to new domains to permit comparisons across diverse contexts including different sociocultural settings. Traditional approaches to gathering data via case reports make it difficult to locate and re-use relevant data despite considerable technological improvements related to the rise of open access and internet-based systems.

Out of recognition of many of these limitations, consensus-based international guidelines have been developed, called the “CAse REport” or CARE guidelines (Gagnier et al. 2013), to increase the completeness in the presentation of published case reports, create more comparability between the data contained in case reports particularly with regard to potential therapeutic interventions and outcomes, and generate more transparency for patients and practitioners, and in turn to inform clinical practice guidelines. When adopted by a journal, these types of guidelines have been argued to be associated with an increase in the completeness of the information published (e.g., Turner et al. 2012) and hence can be viewed as critical pragmatic constructs.

The CARE guidelines are a 13-item checklist outlining basic reporting requirements for published case reports, provided in a structured manner. The key goal is to increase completeness and transparency in published case reports. The authors stress that they “attempted to strike a balance between adequate detail and the concise writing that is one of the appealing characteristics of a case report” (Gagnier et al. 2013, 4).^4^ As discussed elsewhere (Ankeny 2017b), these guidelines are extremely revealing with regard to the underlying epistemology of case reporting particularly in the current era of dedicated journal outlets which have considerable investment in establishing case reports as a valid form of evidence. For the purposes of this paper, I do not analyze them in any detail particularly because their promulgation has been quite recent but do use some of the issues highlighted in the guidelines in my analysis of re-use patterns.

## Detecting Patterns and Themes in Case Reporting and Re-use

### Broader Patterns of Re-use

One of the main potential benefits of publishing case reports (and providing the necessary infrastructures to make them more accessible) is so they can be re-used by others who come across similar phenomena particularly in clinical settings, or so that the observational data can be used as the basis for initiating various types of research. Hence it is useful to look at the broader patterns of re-use to get a sense of the uptake of medical case reports.

More generally, it must be noted that citation analysis may severely underestimate the impact of clinical-oriented research in certain fields particularly in comparison to basic research (e.g., Van Eck et al. 2013) and case reports specifically are cited at a negligible rate compared to other types of publications (Patsopoulos et al. 2005). However for the purposes of this paper, a focus on published literature is appropriate because I am primarily interested in explicit re-uses of data captured in case reports; note that this approach of necessity will fail to capture negative instances, that is, where a case report was accessed and utilized but found not to be relevant to the current problem or phenomenon under examination, in similar ways to the type of publication bias that has been well recognized with regard to negative results (e.g., Kicinski et al. 2015*).* Article usage statistics (available via many journals and databases) might well provide more accurate quantitative information that could be compared across case reports but would fail to allow any assessment of whether or how a case report is being re-used.

Tracking re-use of medical case reports is plagued with technological difficulties, particularly when assessing case reports via citations across all types of journals and medical subfields: for instance although PubMed^5^ indexes nearly half of the journals that publish case reports, excludes most that are likely to be predatory journals (Akers 2016), and provides a “case reports” filter, it does not allow analysis of articles by number of citations (similar limitations occur with Embase, another major medical database). An additional issue is that there are inaccuracies in the tagging of publications as “case reports” (see note 6 below for a rough estimate of the rate of inaccuracy). Even tracing case report patterns by journal by focusing on the dedicated journals is complicated by the fact that several major case report journals have changed name over time and full datasets are thus not readily available.

Hence I used two strategies to analyze case reporting and re-use over the past 25 years: (1) a broader strategy allowing general patterns of re-use (using citations as a proxy) to be visualized; and (2) a more specific strategy focused on highly cited case reports. The temporal window of 1997–2017 was selected to permit inclusion of both the newer journals focused on case reports as well as more traditional journals which publish case reports; it also allows medium- and longer-term re-use to be tracked, since as the analysis reveals, re-use often only occurs over considerable periods of time.

For the first broader search, Web of Science was utilized using a case report focused strategy for medically related fields^6^ to extract data for the years 1997–2017, which generated a total of 108,348 case reports. Just over 30% of these reports have no citations to date, and just over 17% have between one and ten citations since time of publication. A second analysis used the Medline subset within Web of Science, to perform a search for case reports for the years 1997–2017.^7^ The results were 102,195 articles, of which only 98 (slightly more than .09%) of the publications verified to be case reports^8^ were highly cited in their respective field as of March/April 2018 (i.e., they received enough citations to place them in the top 1% of their academic field based on a highly cited threshold for the field and publication year); of these publications, only four were published in the past 2 years and received enough citations to place them in the top 0.1% of papers in their respective academic field. Thus these findings echo previous analyses of the relative neglect of uptake of case reports, but do permit us to focus on those that may have resulted in important instances of re-use.

The highly cited case reports do share certain characteristics: first, they tend to appear in highly popular, general medical journals (e.g., *The New England Journal of Medicine),* which have extremely large readerships. They also cover one of three main topics broadly defined, namely non-randomized and non-controlled trials of experimental drugs or therapies on individuals or very small groups of patients, often on a compassionate or emergency basis; epidemiological or other features of emerging or novel diseases that are typically infectious in nature; and characterization of underlying mutated genetic sequences of disease-related phenotypes or processes at other levels (such as tumors). Less frequent topics include adverse effects of or reactions to therapies of various types; reporting of new illegal drug use and effects; and longer-term outcomes of novel surgical procedures, particularly organ and other transplants. Despite all of these publications being considered to be highly cited, there is no particularly robust correlation between year of publication and the number of citations, and the range in the number of citations is large, from nearly 1500 for a 2011 paper on using modified T-cells to treat leukemia, to 15 for a 2017 paper published in a more narrow subfield, toxicology, focused on episodes of intoxication via a new synthetic opioid.

Although these broader trends give us hints about how data can journey to new domains via case reports, more qualitative analysis helps to reveal precisely what travels from early stage case reports and what roles such data journeying serves. Hence in the following sections, a series of highly cited case reports are analyzed to provide insights into the valuing of data captured by case reports and what factors are associated with re-use. I have opportunistically selected two case reports to explore which have particularly interesting patterns of data re-use but have attempted to represent two of the main types of case reporting captured in the quantitative analysis above.

### Case Reports on Infectious Diseases

One key role played by case reports is to draw attention to emerging or novel infectious disease processes: in recent years, occurrences of Zika, Middle East Respiratory Syndrome (MERS), Ebola, and Influenza A have been described via case reporting, with attention to a range of aspects of the phenomena under study. These case reports often contain important data that then can journey rapidly from their location of creation and reproduce faithfully, as long as certain features are in place.

For instance, a case report (Gao et al. 2013) of three observed human fatalities related to infection with a new form of the avian influenza A virus (H7N9) in Shanghai, China was among the most highly cited (1247 times) in the data set above, as the initial publication relating to what subsequently became a pandemic. Previously the transmission of H7 viruses to mammals had been rarely reported in Asia, human infection with the N9 subtype had not been documented anywhere, and these types of infections had rarely been fatal or as severe as in the patients who presented for care in Shanghai. The case report summarizes the typical information about the patients, including demographic and epidemiological characteristics, particularly those associated with pre-existing conditions likely to have depressed their immune systems as well as potential contact with chickens; the complications, treatment, and clinical outcomes of the patients; and detailed analysis of the characteristics of the virus isolated from the patients.

In conclusion, the authors (many of whom have numerous previous publications on different forms of epidemic influenza particularly in China) make an urgent call to others in the medical field: “We are concerned by the sudden emergence of these infections and the potential threat to the human population. An understanding of the source and mode of transmission of these infections, further surveillance, and appropriate counter measures are urgently required” (Gao et al. 2013, 1896). Among the key points discussed is whether this novel version of the virus occurred within these human hosts or was directly transmitted by birds, with the latter said to be the preferred explanation, particularly based on genetic sequencing and other forms of analysis. However a critical point made in the case report is that influenza surveillance of birds, swine, and humans is limited in China and nearby countries, which makes it very difficult to provide an answer to this question.

With regard to the processes associated with data journeying, a few critical points are notable. First, the initial journeying of the data from the clinical setting to the printed case report (and hence to them becoming available publicly on a global basis) occurred over a highly compressed time period^9^: the patients were seen between mid-February and the end of March 2013, and the case report was published online in mid-April 2013.^10^ Subsequently when published in print in mid-May, it was accompanied by a high-profile editorial by researchers at the US Centers for Disease Control which lauded the authors of the case report for the speed with which the virus was identified and whole genome sequences of it made available, particularly given the global public health issues raised (Uyeki and Cox 2013, which echoes an earlier editorial in *Nature* in April, Anonymous 2013), which was important because of the lack of transparency that sometimes had occurred in the context of past epidemics in China (e.g., with reference to SARS, see Knobler et al. 2004). These factors underscore that the speed with which data from a case report journeys and the extent to which it travels (i.e., how often it is picked up by others reporting research and whether it reaches a global audience) is directly related to a number of factors including the perceived usefulness of the original case report in terms of the data contained within it and the potential threat posed by the condition(s) described, both of which are common in infectious disease related case reports.

Second, data within case reports are more likely to be re-used if they relate to multiple research methods or fields. For instance, the editorial cited above underscored many other critical points raised by the case report, namely that some of the sequence data suggested that this virus was likely to result in asymptomatic or mild avian disease, and thus had the potential to generate a silent widespread epizootic epidemic in China and neighboring countries. Many of the subsequent publications citing the original case report explore these types of issues (e.g., Xu et al. 2013). In addition, in the 6–12 months after the original case report, various members of the research team published more detailed reports (sometimes as research letters, presumably in order to get them published quickly given the urgency of what was quickly becoming a public health crisis)^11^ in high-profile outlets, such as on the biological features of the virus, epidemiological surveillance, and tracing the genesis of the infection via various types of birds (e.g., Lam et al. 2013) which helped to widen the exposure of the original publication particularly in fields beyond infectious disease. Hence various types of data originally contained within the original case report journeyed without necessarily being closely connected to the initial case report. Examples include numerous publications related to technology development such as new methods for real-time detection of infection (e.g., Zhu et al. 2013).

Finally, the “call to arms” for more surveillance and reporting in the case report (and associated publications such as the accompanying editorial) resulted in numerous publications about additional instances of the disease, as well as having clear public policy implications, which also appears to be a mark of a case report from which data are likely to journey. Thus potential wider relevance along with “actionability” of data (see Ramsden in this volume) is often associated with wider patterns of journeying. For instance following the case report and in part based on its findings, H7N9 influenza was established as a notifiable infectious disease in Taiwan which experienced a spike of cases amongst travelers returning from China soon after the initial outbreak in China (TCDC 2013). As underscored in a paper citing the original case report, one of the lessons to be learned from this case report is more generic, and relates to the importance of this type of data having a way to journey outward, particularly given certain tendencies reinforced in medical training: “Instead of recognizing that billions of people worldwide are exposed to important and emerging infectious diseases, our training has relegated this topic mostly to ‘tropical medicine’ or public health or labelled the threat as a ‘zebra’ item” (McFee 2013), referring to the medical training adage that “if you hear hoofbeats, think horses, not zebras” (see Hunter 1996; Wright and Kouroukis 2000). Given increased globalization together with the emergence of various serious health threats, some “zebras” are now critically important, and there is a critical need for pandemic preparedness. Thus these sorts of public health emergencies require not only rapid data collection and analysis, but also data sharing and feedback (Uyeki and Cox 2013; see also Lurie et al. 2013) via “data journeying” particularly in conceptual terms. Case reporting provides a clear mechanism for these processes to occur, especially where detailed data are provided in case reports that are useful for epidemiological tracking and related processes (Anonymous 2013).

### Case Reporting of Adverse Effects

Another key category of case reporting relates to adverse or unexpected effects particularly of commonly utilized treatments or drugs. Consider a highly cited case report detailing two fatalities and one life-threatening incident in young children related to consumption of codeine for pain relief after adenotonsillectomy for obstructive sleep apnea syndrome (Kelly et al. 2012). The Canadian team proposed that where the surgery has not resolved the sleep apnea, morphine is particularly dangerous as it may further worsen the respiratory condition, can be fatal in cases where children have a certain genetic allele that can lead to a toxic accumulation of morphine exceeding therapeutic levels, and is of particular concern in individuals of North African descent where the mutation is more common (occurring in 30% of the population).

Some members of the team (together with the chief coroner for the province) had previously published a letter in 2009 focused on a single case similar to the 2012 series which documented the death of an otherwise healthy 2 year old with functional duplication of a particular genetic allele known to be associated with increased rates of conversion of codeine to morphine and which may have contributed to respiratory depression and death, in concert with other factors (Ciszkowski et al. 2009). In this letter, the authors declare that “given the polymorphic nature of codeine metabolism and the fact that adenotonsillectomy does not reverse all cases of obstructive sleep apnea, codeine cannot be considered a safe outpatient analgesic for young children after adenotonsillectomy.”

Tracing the citations to the 2012 case report reveals several key themes: first, the uptake of the 2009 letter (and the data contained in it) was much more limited, based on citation patterns, than the case report which appeared later, despite both appearing in very high-profile medical journals (*The New England Journal of Medicine* and *Pediatrics* respectively). However there are several reasons which seem to be correlated with this difference, notably that the 2012 case report was in fact peer-reviewed and detailed multiple instances of the observed phenomenon. Description of multiple occurrences of a phenomenon appears to result in the case report and the data contained in it being valued more highly, likely because it is viewed by readers as providing more or more robust evidence especially because other underlying factors can be ruled out; even if three cases may seem to many to still be anecdotal, in this example multiple cases appear to have resulted in more re-use of data and of the case report itself, at least in the form of citations.

An additional trigger which contributes to wider recognition and re-use of case reports is whether the observed adverse effects come to be formally certified, such as in recognition by regulatory authorities or professional organizations. In the current case, during late 2011, the Patient Safety and Quality Improvement Committee of the American Academy of Otolaryngology–Head and Neck Surgery (AAO-HNS) had become concerned about adverse events, particularly respiratory depression, after adenotonsillectomy and conducted a nationwide, anonymous survey of otolaryngologists about such events (Racoosin et al. 2013). By August 2012 following an evaluation of the safety of use of codeine in children including a comprehensive review of the literature and case reports submitted to the US Food and Drug Administration (FDA)’s Adverse Event Reporting System, the FDA issued a press release and drug safety communication warning of the risk of respiratory depression and/or death following the use of codeine after tonsillectomy. Its review found 13 cases, including 10 deaths and 3 cases of life-threatening respiratory depression associated with codeine use during the period 1969 and 1 May 2012 (including the original case reports). The issuing of the FDA advisory is correlated with a sharp increase in citations to the 2012 report, likely simply out of increased awareness of these issues, with many of the publications exploring implications of these findings for codeine use in children in this or other types of care settings.

In addition, the scale of the potential for adverse effects clearly contributes to the re-use of case reports. Although the complication in the case at hand is likely rare, it has the potential to affect a significant number of children given the huge number of adenotonsillectomies performed per year, about half a million annually (Cheng and Sobol 2013). Case reports are more likely to be viewed as oddities or mere anecdotes if they seem to have very small-scale effects, in which case they are obviously not particularly ripe for re-use of the data contained within them.

A final factor about whether data contained in case reports about adverse effects are subsequently re-used seems to be related to whether they align with other broader epistemological understandings or trends in patient care, public health, or other types of medical practices (again here compare the chapters by Cambrosio et al. and Ramsden with particular attention to the idea of actionability of data especially in clinical research practices). There are at least two potential ways in which these issues are likely have been in play in this example: first, as noted in a Perspectives piece published in *The New England Journal of Medicine* following the FDA warning, increased awareness of what they term “the value of both personalized medicine and the reporting of rare adverse outcomes” (Racoosin et al. 2013, 2155) has resulted in more attention to and publicity about such adverse effects. In other words, the genomic turn of the early 2000s has resulted in greater awareness of genetic diversity including mechanisms relating to drug reactions, and greater abilities to provide alternative clinical treatments. These claims are substantiated in the types of articles citing this case report, many of which make reference to the need for more precise methods to determine optimal approaches to pain control, particularly with young children post-adenotonsillectomy, and some of which position these claims explicitly within the emerging field of pharmacogenetics (e.g., Lee et al. 2014; Smith et al. 2018).

But a second likely trigger of the patterns of re-use observed relates to the increasing awareness of the so-called “opioid epidemic” in the 2010s, especially in the United States.^12^ Due to increases in opioid-related addiction, overdoses, and deaths, opioid use came to be viewed as a public health crisis in this period, in part related to illegal drug use but also in concert with over-prescription of legal pain medications including oxycodone which is chemically and otherwise similar to codeine. Thus in the re-use of data from the original case report, we find it cited simply as evidence of the potential dangers of codeine use in articles more broadly exploring the potential benefits and dangers of prescribing it not only for children (e.g., Carter et al. 2013: Martin et al. 2014) but in certain groups likely to be more at risk such as immigrants (e.g., Ray et al. 2014, which in fact observed no increased risk in these groups despite language and genetic differences). Further, due to subsequent changes in the way the FDA classified (“scheduled”) hydrocodone combination products in 2013, several of the publications (e.g., Fleming and Wanat 2014) emphasize the potential dangers of codeine-based products for pain management, in part out of recognition that there would be a tendency to increase use of these products as these remain accessible at levels requiring less approval processes. Hence as this case report shows, re-use of data can become quite loose and its journeying more akin to wandering where some part of the case report proves to have much broader relevance, and particularly where there are practice and public health implications.

## Conclusions: Implications for Understanding How Data Journey

What makes data more likely to journey beyond their original case reports? It is clear that a few factors can be identified; though these are neither necessary or sufficient, they do provide some marks that assist us with understanding the potential epistemological value of case reporting and the data contained within them. First, case reports that have implications well beyond their immediate domain are likely to be published in general medical journals which allows them to be read much more widely, and hence to much more easily be conceptualized as having broader relevance. Second, the data contained in case reports tend to journey when they have content with broader implications well beyond the case report at hand, and particularly when the data have evidential value for topical or even urgent issues, particularly those arising in public health. Any potential for wider applicability may well not be explicitly detailed in the original case report, but can be spurred on by additional factors, such as relevance for policy, uptake and endorsement by professional organizations or governmental authorities, description in other contexts such as framing editorials accompanying the case report, and so on.

Third, use of multiple research methods or concepts from diverse subfields within medicine can expedite the journeying of data within a case report into a range of types of journals and allow the data to journey well beyond their original context. Thus larger teams of authors are often common in the most highly cited case reports, likely in part because diverse expertise is necessary for case reports that bring together different types of data, but this pattern in turn seems to support greater potential for the data to journey more widely. Finally, data from case reports tend to journey where there is alignment with broader epistemological understandings or agendas within medicine: for instance the turn toward genomics in the 2000s resulted in journeying of data associated with numerous case reports related to unusual phenotypic disease patterns or adverse effects to other contexts, notably to publications detailing more fundamental biomedical research to determine the genetic basis for these patterns or effects.

What can be said about the efficiency of the journeying of data from case reports? The empirical data and qualitative analysis presented above reveal that the speed with which data from a case report journey and the extent to which they travel is correlated not only with the perceived usefulness of the original case report in terms of the original data contained within it (as would be expected) but also by the potential threat posed by the condition(s) described: so infectious disease-related case reports often are urgently reported and data from them picked up elsewhere. In addition, as occurred in the case report on the adverse effects of codeine in young tonsillectomy patients, data associated with case reports where broader implications subsequently come to be recognized (e.g., for postoperative pain control or even pain control in general in this example) have their journeying expedited by their application in these broader contexts.

These issues related to journeying take us back to the various efforts to standardize case reporting: why bother limiting data captured by case reports to certain categories when our technological infrastructures in fact might permit us to “write down everything,” and in principle create more potential for journeying? One part of the answer clearly relates to the requirement that case reports be useable by medical practitioners who are both the authors of the guidelines and many of the likely users (and re-users) of the case reports and the data contained in them: not all data that might be captured and packaged in a case report are of equal relevance, which can be seen in the factors more closely associated with journeying outlined above. Thus new efforts at highly structured guidelines about what must be included impose a certain rigor to what is thought to be essential for understanding a case report and for re-using the data contained in them to identify similar cases or other domains where the data might have relevance. Though in some sense it is technically possible to include absolutely all data (or many more pieces of data than currently contained in case reports), to do so would undermine the structures (narrative and otherwise) that form the basis for what the case report is a case *of,* and hence place limits on the abilities of practitioners to re-use it.

In addition, these guidelines have certain merits beyond mere standardization for ease of re-use of case reports and the data within them: at a deeper level, they constitute a line of attack on traditional assumptions regarding what types of data are valued and under what circumstances. Case reporting in a standardized manner reinforces the value of data derived from individual case reports and helps to establish methods for consistent re-use. These types of guidelines also underscore how data can serve evidence in these sorts of observational settings that previously have been assumed to be unable to be systematized in any significant ways, particularly as compared to RCTs and other experimental methodologies. As the authors note, what is most critical is that case reporting be made more precise, complete, and transparent (Gagnier et al. 2013), which no doubt is correct. However as this paper has shown, there are deeper epistemic issues underlying the re-use of case reports and the journeying of the data within them, and these guidelines have the potential to allow both creators and users to be reflective about both the potential (and limitations) of case reporting, particularly in the context of re-use.

Exploring the effective journeying of data contained in case reports together with efforts to standardize the presentation of data are important parts of developing deeper understandings of appropriate, effective, and rigorous ways of using observation-based methodologies in the biomedical sciences and other fields that rely on such approaches, given that these have been largely neglected, for instance in medicine due to the rise of EBM and related approaches in which data are relatively easy to systematize (cf. Tempini and Teira in this volume on the difficulties of circulating data in other settings). As the guideline authors state, “When it becomes clear how new data contributes to evidence, the stewardship needed to produce high-quality data will become more rewarding and our attitude toward ‘observation’ will shift…This will transform how we think about ‘evidence’ and revolutionize its creation, diffusion, and use—opening new opportunity landscapes” (Gagnier et al. 2013, 5). How these types of data journey faithfully and efficiently in a variety of contexts and hence come to be valued as a form of evidence warrants further exploration.

## Footnotes

1 See Beatty 2016, 2017 on issues relating to contingency as well as what makes something narrative-worthy.

2 On similar processes in natural history, see Terrall 2017.

3 On narratives in case reports, see especially Hurwitz 2017; on narrative in medicine, see Gygax and Locher 2015; Hurwitz and Bates 2016.

4 This structure also aims to capture “useful” information including that required by the U.S. Department Health and Human Services to demonstrate so-called “meaningful use” of certified electronic health records, which in turn is required by some private insurers for physicians to for health providers to quality for certain types of performance incentives. Although intriguing to consider the epistemological impacts of these social and financial incentives, an analysis of the interplay between these requirements and the content of case reporting guidelines is beyond the scope of this paper.

5 PubMed is a free search engine which primarily allows access to the MEDLINE database of references and abstracts on life sciences and biomedical topics, which in turn is managed by the United States National Library of Medicine at the National Institutes of Health as part of the Entrez system of information retrieval.

6 This search was performed on 14 November 2018 by utilizing Web of Science Core Collection to search for all items tagged as topic = “case report∗” for the years 1997–2017, which generated a list of all items tagged as case reports from more generalized journals. This set was then supplemented by inclusion of all publications in case report-focused journals for the same time period (identified by explicit inclusion of “case report” or similar in the journal titles, and drawing on the list published in Akers 2016). These sets were combined and then narrowed to include only those with topic = “human∗” or “patient∗”, and by excluding publications coded to non-medical categories. The publications were then run through Clarivate InCites to obtain rates of citation.

7 This search was performed by selecting “case report” in the document type field for the years 1997–2017, then limiting to core clinical journals and to humans (17 July 2018).

8 The original set that was automatically generated on 17 July 2018 included 118 articles, of which 20 (17%) were determined not to be case reports based on manual review of abstracts; some appeared to be review articles that had been mistagged whereas others were very large observational studies that strictly speaking would not typically be considered to be serial case reports but which some journals nonetheless place in their “case report” sections.

9 Case reports typically have longer gestation times between clinical observation, laboratory analysis, and other processes, and actual publication, even when focused on similar public health related issues: see for instance Colson et al. 2010 on a small case controlled study within a single family on the transmission of hepatitis E via figatellu, a traditional pig liver sausage widely eaten in France and commonly consumed raw, where initial observations and data collection occurred in 2007–9 but which was not published until 2010; nonetheless this case report also is among the most highly cited in its field.

10 Although beyond the scope of this paper, it is worth noting that formal mechanisms such as infectious disease reporting and more informal mechanisms such as media coverage can help data in a case study to journey. According to the journal *Nature* (Anonymous 2013), China reported the H7N9 outbreak to the World Health Organization (WHO) on 31 March 2013, and simultaneously published the genomic sequences of viruses from the three human cases on the database of the Global Initiative on Sharing Avian Influenza Data (GISAID). It also shared all of the sequences with the WHO, and live virus with the WHO and other laboratories. In addition, the Chinese media reported new cases on a daily basis and discussed H7N9 fairly openly, with Chinese President Xi Jinping publicly calling for an effective response, noting that the government should ensure release of accurate information about the outbreaks.

11 Self-citations are common among citations to previously published case reports, and are difficult to systematically eliminate from larger datasets when mapping patterns of re-use.

12 Even using the terminology of “epidemic” in this context raises a range of historical, political, and sociological issues, but this issue is not a main focus in this paper; for discussion, see for instance Green et al. 2002; Martin and Martin-Granel 2006.
